# Quantitation of Cannabidiol (CBD) in brain regions and plasma following intranasal administration of a CBD nanoformulation

**DOI:** 10.1186/s42238-025-00308-5

**Published:** 2025-08-22

**Authors:** Gunjan Upadhyay, Oksana Fihurka, Pranav Patel, Juan Sanchez-Ramos

**Affiliations:** 1 Department of Neurology, University of South Florida, 13220 USF Laurel Drive Rm 4105, Tampa, FL 33612 USA; 2 SGN Nanopharma, Inc, 3720 Spectrum Blvd., Tampa, FL USA

**Keywords:** LC–MS/MS, CBD, 7CBD-COOH, Intranasal administration, Nanofomulation, Δ^**9**^THC, Pharmacokinetics, Blood–brain barrier

## Abstract

**Background and objective:**

Delivering therapeutic drugs to the brain for neurological disorders remains challenging due to the restrictive nature of the blood–brain barrier (BBB). Intranasal (IN) nanoparticle delivery may enhance the bioavailability of lipophilic cannabidiol (CBD), addressing limitations associated with systemic administration.

**Methods:**

Further optimization of nanoparticle properties is necessary to enhance brain uptake and therapeutic potential for neurological disorders. Following IN administration of the nanoformulation, C57BL/6 male mice (3–6 months old, *n* = 4/group) were euthanized at 2, 4, and 8 h. Plasma, olfactory bulb (OB), hippocampus (HP), striatum (STR), and cortex (CTX) were collected and analyzed for CBD and 7-COOH-CBD using liquid chromatography-mass spectrometry (LC–MS). Two-way analysis of variance with Tukey’s multiple comparisons was used for statistical analysis.

**Results:**

CBD levels in the brain peaked at 4 h (5788 ng/mg), while 7-COOH-CBD reached its highest concentration at 2 h (3080 ng/mg). In plasma, maximum CBD levels were detected at 4 h (797 µg/mL), whereas 7-COOH-CBD peaked at 2 h (893 µg/mL). Despite measurable brain penetration, only 0.12% of the administered dose reached brain tissue, with 15.94% retained in plasma.

**Conclusion:**

This is the first study to provide the quantification of CBD and its 7CBD-COOH in various brain regions following IN administration of a CBD nanoformulation. While the approach facilitated brain delivery, overall bioavailability remained low. The use of four mice per group is a limitation that may impact the internal validity of these findings. This study aimed to develop a novel hydrophilic CBD nanoformulation for IN delivery and quantify its distribution and its major metabolite, 7-carboxy-cannabidiol (7CBD-COOH) in distinct brain regions and in plasma of mice.This methodology has the potential to overcome the limits of conventional CBD administration, providing a more effective treatment strategy for targeting brain diseases.

## Introduction

Cannabidiol (CBD), a non-psychoactive cannabinoid, has gained significant attention in recent years for its therapeutical applications in various neurological disorders (Yau, et al., 2023; Pozo, et al., 2024). CBD at high doses has been reported to be effective in managing some forms of epilepsy, but it is also very promising for chronic pain and ulcerative colitis (Schouten, et al., 2023; Zhang, et al., 2024; Muresan, et al., 2023). In addition, CBD activates peroxisome proliferator activated receptor gamma (PPARγ) and also has been shown to exhibit neuroprotective effects by modifying neuroinflammation (Frederico Gava, et al., 2024).

Intranasal (IN) administration offers a promising route for CBD delivery due to its potential for enhanced bioavailability and direct access to central nervous system (CNS). However, optimizing CBD’s pharmacokinetic profile, particularly its brain penetration and distribution within the CNS remains a challenge.

Nanoformulations, due to their small size, have emerged as a strategic approach to improve drug delivery. Especially, a Nanoformulation of CBD exhibited a 419.64% higher in vitro drug release and a 76.17% higher *ex-vivo* nasal permeability compared to CBD alone due to the improved bioavailability, controlled release, stability and efficacy (Ahmed, et al., 2022). Furthermore, IN administration of CBD-infused hydrogels has shown promising results in reducing inflammation, by reducing the concentrations of Tumor Necrosis Factor-α (TNF-α) in the brain, suggesting therapeutic potential for post-traumatic stress disorder (PTSD) (Pang, et al., 2021).

While CBD has shown efficacy in managing certain types of epilepsy, chronic pain and ulcerative colitis, its therapeutic potential is likely influenced by its pharmacokinetic properties including absorption, distribution, metabolism and excretion. The route of administration significantly impacts CBD’s brain penetration and overall effectiveness (Poklis, et al., 2010). For example, CBD levels in brain reached 3.9 ng/g when administered by inhaling smoke from plant material containing 0.93 mg of CBD (Poklis, et al., 2010). And hence IN delivery using CBD nanoparticles may offer improved absorption into the blood stream and potentially greater CNS access by utilizing nasal cavity’s thin capillary epithelial cell lining (Eydelman, et al., 2023; Upadhyay, et al., 2023).

This study aimed to address the need for optimized CBD delivery to the brain by developing a novel hydrophilic Nanoformulation. This formulation was designed to reduce rapid systemic absorption and enhance direct transport to he brain via olfactory, trigeminal and perineural pathways (Upadhyay, et al., 2023). We analyzed CBD and it’s major metabolite, 7-carboxy-cannabidiol (7CBD-COOH) in the brain region and plasma samples from mice following the IN administration of this CBD nanoformuation. By characterizing the pharmacokinetic profile of this formulation, we sought to gain insight into its potential for therapeutic applications and compare its distribution and metabolism to our previous findings on Δ^9^Tetrahydrocannabinol (THC) (Upadhyay, et al., 2023). This approach leverages the advantages of the IN delivery, such as a faster onset of action and improved bioavailability due to the avoidance of first-pass hepatic metabolism (Polidoro, et al., 2022; Marusich, et al., 2023).

## Materials and methods

### Preparation of nanoformulation of CBD

About 2.5 g of CBD (Mile High, lot No- IL2002I-005B) was weighed in a glass beaker. To it 0.5 g of Ethanol (Decon- labs, lot no-102214) and 6.5 g of Croda GTCC oil (Croda, lot no 42731) and 1 g of Polysorbate-80 (Acros, lot no-A0120589) were added. This mixture was sonicated till the CBD dissolved completely and a clear solution was formed. This was an oil phase. In another beaker, 1 g Beta-cyclo-dextrin (BCD) purchased from Acros (lot no A0382219) was dissolved in 38 g of Milli-Q water. This was the water phase. The water phase was slowly added to the oil phase drop by drop while stirring using an electric stirrer for about 15 min to make water in Oil (W/O) emulsion. The pH of this solution was set to be around 7 using dilute sodium hydroxide solution. This emulsion was passed through the homoginzer (make-Avestin, Emulsiflex C3) for 3 cycles at 10,000–15,000 psi of pressure. The particle size of this formulation was checked using Malvern Zetasizer Nano ZS and found to be an average size of less than 150 nm. This formulation contained 5 mg CBD (Upadhyay, et al., 2023). Cannabidiol (CBD), Lot no- FE10071912, Cannabidiol- Deuterated (CBD-D_3_) Lot no- FE12121902, Cannabidiol-7-oic acid (7COOH-CBD) Lot no- FN04152105, Cannabidiol-7-oic acid-Deuterated (7COOH-CBD-D_3_) Lot no- FN08122004 and Ammonium format Lot no- BCCG7486 were ordered from Sigma Aldrich.

### Animal care compliance

This study followed ethical guidelines for animal research, including the"Guide for Laboratory Animal Care"(8th edition, 2011) and the American Veterinary Medical Association (AVMA's) Euthanasia Guidelines (2013 edition). All procedures were approved by the University of South Florida's animal care committee. The study used male mice (3–6 months old) of the FBV/N strain. These were regular, non-genetically modified littermates of YAC128 (stock no. 004938) male mice available from Jackson Laboratories (Bar Harbor, ME, USA). The university's animal facility bred and maintained the mice used in the experiment. The mice had regular housing conditions with constant access to food and water.

### Study design

**Fig. 1:**
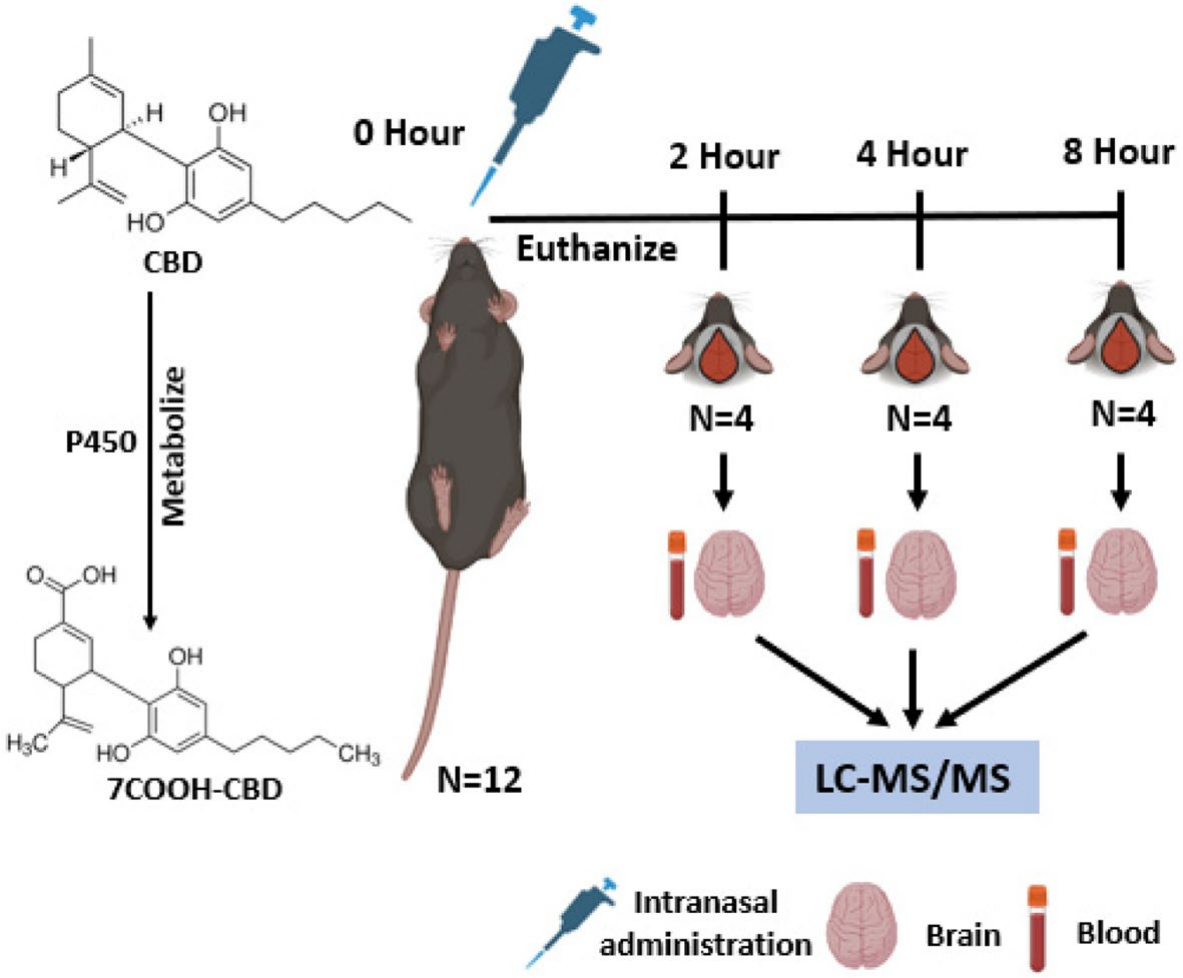
Study design

Twelve mice were given an IN dose of the 5% CBD nanoformulation**.** Ten instillations of 5 µL were given to each nostril for a total of 50 µL, resulting in 182 mg/kg body weight. Mice were euthanized at the end of 2, 4 and 8 h and tissues and blood were collected (Fig. 1).

### Intranasal (IN) administration

Isoflurane was used to lightly anesthetize mice before administering the IN medication. The nanoformulation was delivered IN to each mouse, which was kept in a supine position with necks parallel to the ground the entire time. Drop by drop, 5 µL of nanoformulation was injected into one nostril; after 15 s, the other nostril received the identical treatment. This was repeated 10 times so a total of 50 µL of the formulation was administered and the mouse was returned to the anesthetic cabinet (Hanson, et al., 2013; Miwa, et al., 2020).

### Necropsy

The mice were euthanized via intraperitoneal injection of ketamine at 2, 4, and 8 h following intranasal administration of 50 µL of a nanoformulation containing 5% CBD. After euthanasia, a subcutaneous injection of heparin (4 IU/g) was administered, and intracardiac blood was withdrawn. Blood samples were centrifugated at 1000 rpm for 5 min at + 4 °C and the plasma was separated. Subsequently, brain tissues were collected, with specific disswction of the hippocampus (HP), corps striatum (STR), cerebral cortex (CTX), and olfactory bulb (OB). All samples were then stored frozen at −80 °C until analysis.

### Statistical analysis

Data is expressed as the mean and standard error of the mean (SEM). Each cohort consisted of 4 mice. Statistical analyses were performed using GraphPad Prism 9. Analysis of variance, Two-way ANOVA (CBD dose and time) with Tukey’s multiple comparisons was used, *p* < 0.05 was considered statistically significant.

### MetaboAnalyst6.0

Pharmacokinetics analysis was done using MetaboAnalyst 6.0. Sample normalization was done by average. Heat maps were created using the ward clustering method with euclidean distance measure. The heat map represents the relative concentration of CBD and 7COOH-CBD across different brain regions and time point. The color scale ranges from blue (representing the lowest detected values) to red (representing the highest detected levels). This relative scale, with negative values indicating concentrations below the average. The statistical significances of the group patterns for Principal Component Analysis (PCA) were evaluated using PERMANOVA.

### Sample extraction for LC–MS

For each brain region- olfactory bulb (OB), hippocampal (HP), stratum (STR), and cortex (CTX), approximately 10–20 mg of tissue was weighed and 1 ml of menthol was added to it. These samples were homogenized in the same solvent to form a uniform mixture. 1 mL of Acetonitrile was added to 100 µL of both blood and plasma samples. To all samples, including the tissue, 1 ng of CBD-D_3_ and 7COOH-CBD-D_3_ internal standards were added. These were vortexed using Thermo Scientific vortex for about 5 min and kept at −30 °C for 15 min to precipitate the proteins. All the samples were centrifuged at 4500 rpm for 15 min and the supernatant was collected in a glass vial and injected into the LC–MS for analysis.

### Chromatographic conditions

3µL of standards and sample were injected into Waters UPLC Class-I, Xevo TQ-S micro mass spectrometer spiked with 100 µg/mL containing internal standards using ACQUITY UPLC BEH C18 column from Waters (100 mm*2.1 mm*1.7 µm). The mobile phase, sample and column temperature, gradient, flow rate, run time, cone gas flow and other chromatographic conditions and mass spectrometer conditions were followed exactly from the previously published manuscript (Malaca, et al., 2021). Mass Lynx software was used for the analysis. Blanks and placebos were injected to make sure there were no false positive results.

### Calibration curve

**Fig. 2:**
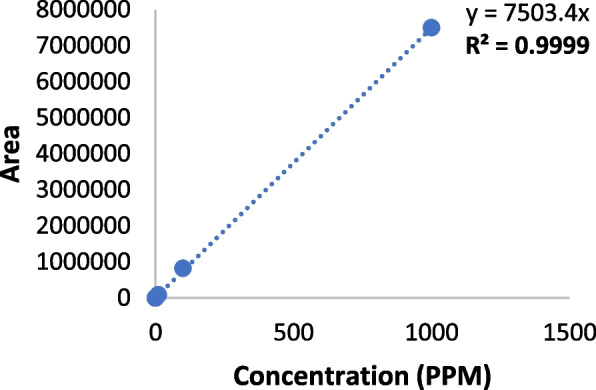
CBD Calibration Curve

**Fig. 3:**
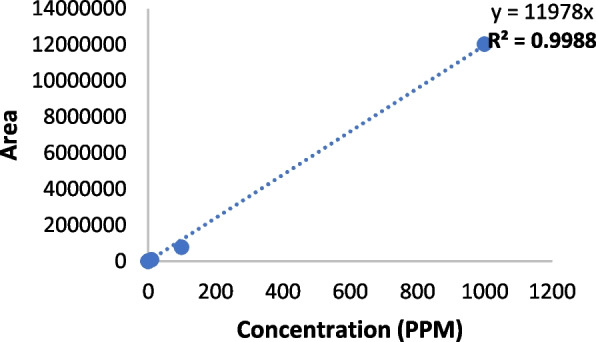
7COOH-CBD Calibration Curve

The calibration curve for CBD was found to be linear for CBD and 7COOH-CBD in the range of 0.001 Parts per Million (PPM) to 1000 PPM. The values for the area are provided for CBD and 7COOH-CBD (Tables 1 and 2). The coefficient of determination (R^2^) value was found to be 0.9999 for CBD and 0.9988 for 7COOH-CBD (Figs. 2 and 3).

**Table 1:** Concentration and area of CBD standard

PPM	Area
0.001	276.3
0.01	340.9
0.1	965.3
1	8186.0
10	83,503.0
100	822,152.9
1000	7,496,111.5

**Table 2:** Concentration and area of 7-Carboxy cannabidiol (7COOH-CBD Standard)

PPM	Area
0.001	750.4
0.01	179.1
0.1	920.2
1	8771.1
10	86,229.7
100	782,325.6
1000	12,020,273.0

## Results

After the dose of 182 mg/kg of CBD, the mice were euthanized at 2, 4 and 8 h and the concentrations were determined using mass spectrometry. The mean values for brain region HP, ST, CTX and OB are provided as nanogram/milligram (ng/mg) of the organ weight and for blood and plasma, the means are expressed in microgram/milliliter (ug/mL) and standard error of the mean (SEM) (Table 3).

**Table 3:** Concentrations of CBD and its metabolite 7-Carboxy cannabidiol (7COOH-CBD) in hippocampus (HP), corps striatum (STR), cerebral cortex (CTX), and olfactory bulb (OB) regions of brain, blood, and plasma at different time intervals

Drug	Organs	Mean 2 h ± SEM	Mean 4 h ± SEM	Mean 8 h ± SEM
CBD	HP (ng/mg)	370 ± 14	569 ± 68	334 ± 152
	STR (ng/mg)	1469 ± 73	1539 ± 142	977 ± 27
	CTX (ng/mg)	231 ± 14	235 ± 68	649 ± 470
	OB (ng/mg)	1809 ± 486	3445 ± 977	2057 ± 745
	Blood (ug/mL)	347 ± 21	578 ± 195	422 ± 41
	Plasma (ug/mL)	469 ± 19	797 ± 89	557 ± 36
7CBD-COOH	HP (ng/mg)	251 ± 21	221 ± 15	169 ± 9
	STR (ng/mg)	222 ± 18	141 ± 26	100 ± 10
	CTX (ng/mg)	1 ± 0.3	1 ± 0.2	0.4 ± 0.1
	OB (ng/mg)	2606 ± 442	2039 ± 231	1668 ± 240
	Blood (ug/mL)	74 ± 2	85 ± 16	47 ± 4
	Plasma (ug/mL)	893 ± 40	708 ± 47	556 ± 30

**Fig. 4:**
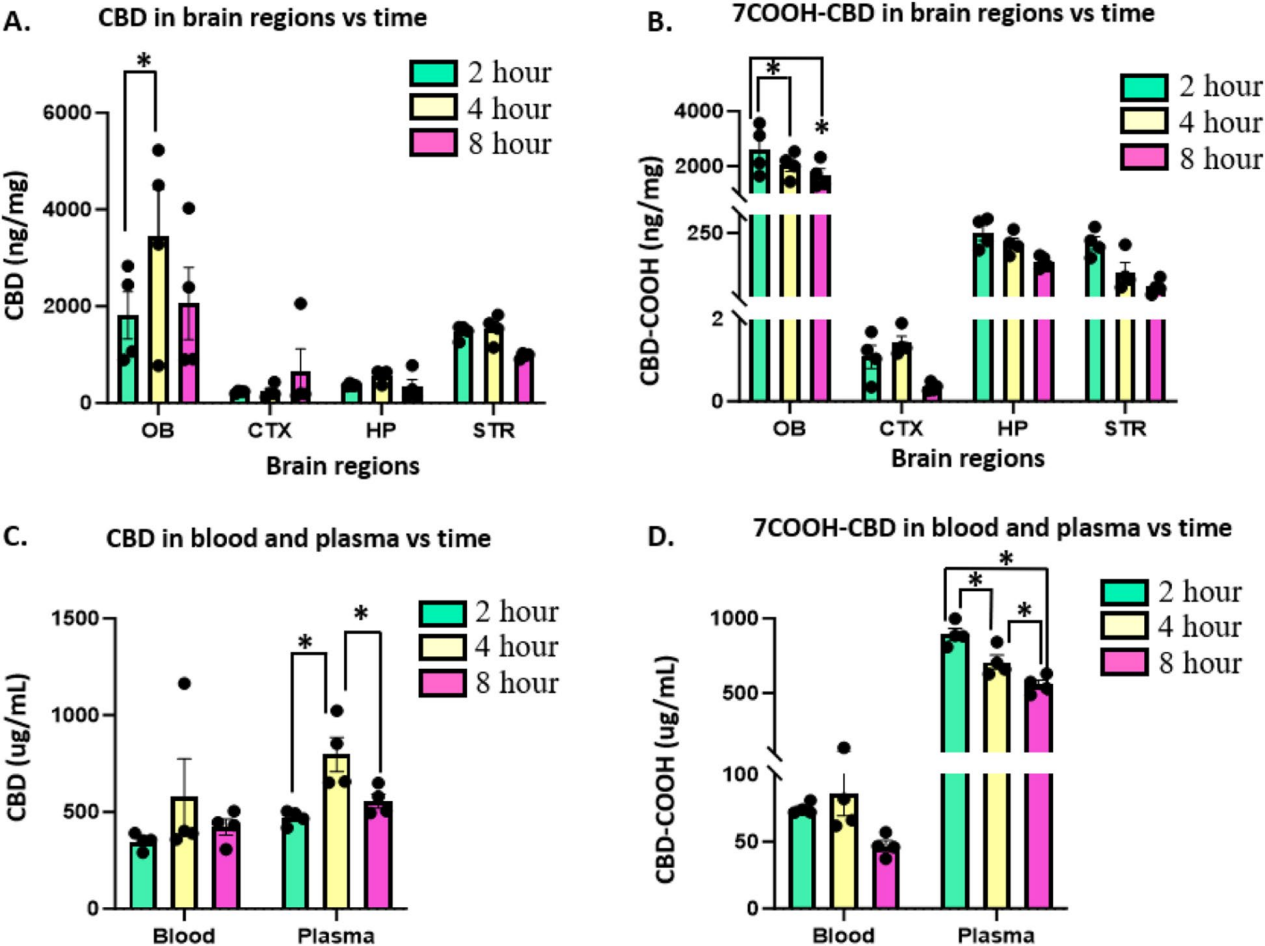
Changes over time of concentrations of CBD and its metabolite 7-Carboxy cannabidiol (7COOH-CBD) following IN instillation in interleaved scatter with bar plots. **A** Changes over time of CBD (ng/mg) in four brain regions hippocampus (HP), corpus striatum (STR), cerebral cortex (CTX), and olfactory bulb (OB). **B** Changes over time of 7COOH-CBD (ng/mg) in four brain regions hippocampus (HP), corps striatum (STR), cerebral cortex (CTX), and olfactory bulb (OB). **C** Changes over time of CBD (ug/mL) in blood and plasma. **D** Changes over time of 7COOH-CBD (ug/mL) in blood and plasma. Two-way ANOVA analysis (brain regions vs time) was performed and * = *p* < 0.05 was used. *N* = 4 mice for 2, 4 and 8 h cohort. Each panel showed the brain region and blood, and plasma accounted for the greatest percentage of the total variance in levels of CBD and 7COOH-CBD. Tukey’s Multiple Comparison reveals significant differences in OB and plasma at different time points (* = *p* < 0.05)

In the brain, the highest concentration of CBD was found at 4-h (3445 ± 977 ng/mg) which was significantly higher than the concentration of CBD in 2 h in OB region. No other statistically significant changes were seen for CBD in the brain. CBD was detected in CTX, HP and STR region as well, with the lowest mean concentration of 231 ± 14 ng/mg, measured in the CTX at 2 h (Fig. 4A). The metabolite of CBD, 7COOH-CBD was statistically highest at 2 h (2606 ± 442) ng/mg in the OB region compared to 4 and 8 h. The concentration of the metabolite was found to decrease at 4 and 8 h in OB, HP and STR and the lowest concentrations (1 ± 0.2 ng/mg at 4 h and 0.4 ± 0.1 ng/mg at 8 h) were measured in CTX brain region (Fig. 4B). In the blood, no statistically significant changes were observed for both CBD and 7COOH-CBD but in the plasma, significantly high concentrations of CBD were found at 4 h, and at 2 h for 7COOH-CBD. CBD and its metabolite levels were highest in plasma (Fig. 4C, D).

**Fig. 5:**
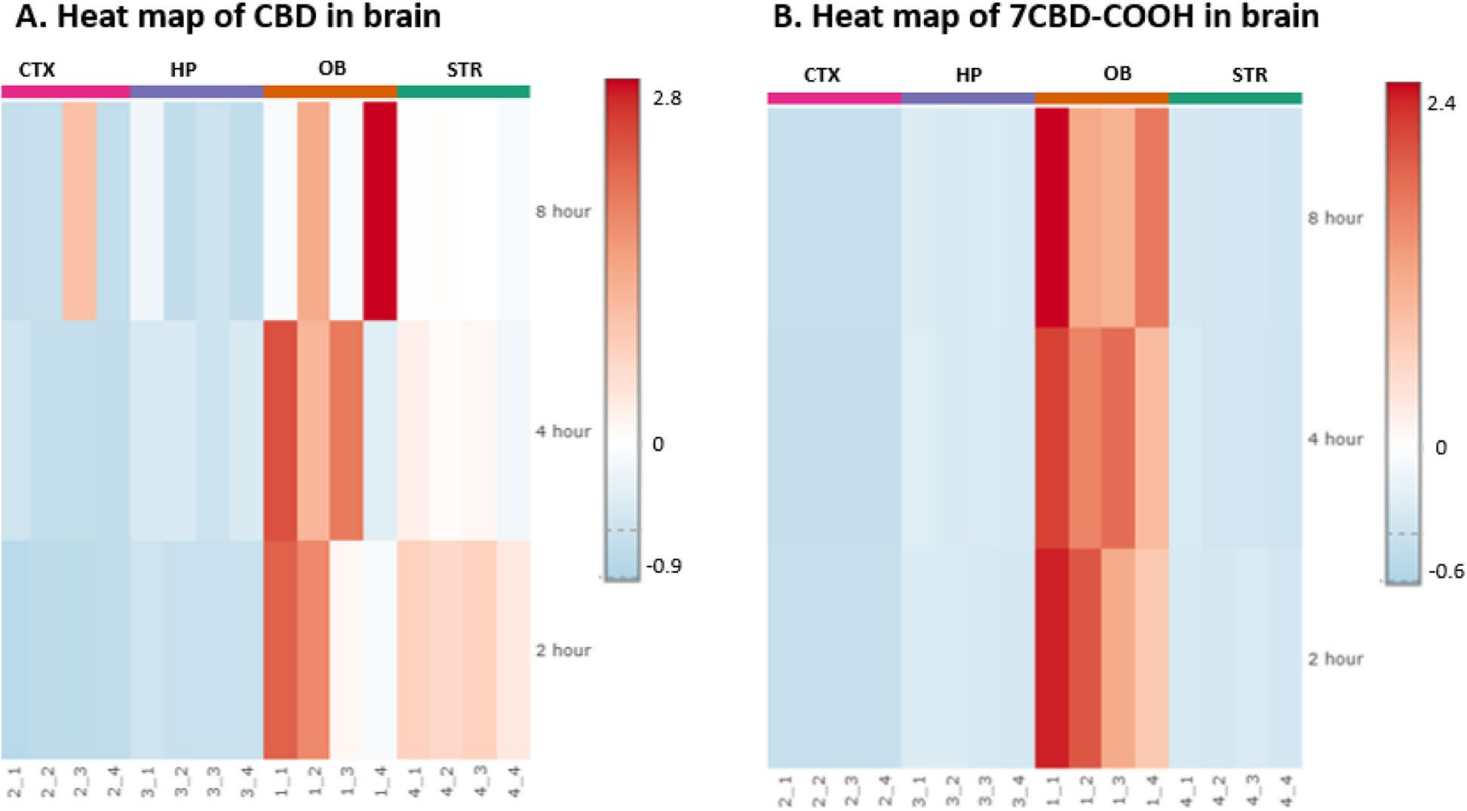
Heat maps showing the relative drug concentration in the hippocampus (HP), corpus striatum (STR), cerebral cortex (CTX), and olfactory bulb (OB) brain regions at 2, 4, and 8 h. The color scale ranges from blue (representing the lowest detected values) to red (representing the highest detected levels). This relative scale, with negative values indicating concentrations below the average for: **A** CBD (**B**) 7-Carboxy cannabidiol (7CBD-COOH)

The Heat map revealed the drug distribution of CBD across various brain regions. The concentrations of CBD in the STR region were highest at 2 h and tended to decrease over the 8-h time interval (Fig. 5A).

**Fig. 6:**
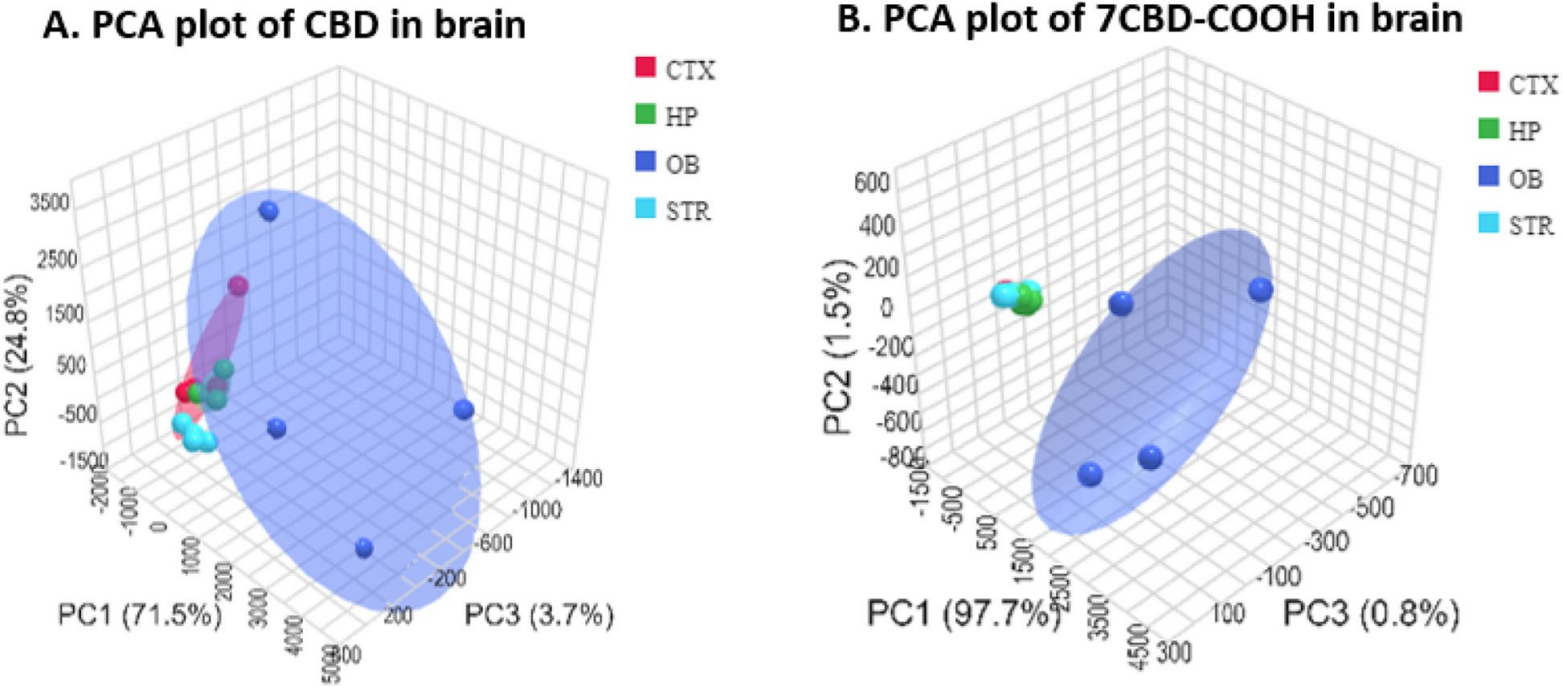
The multivariate principal component analysis (PCA) showing drug distribution in hippocampus (HP), corpus striatum (STR), cerebral cortex (CTX), and olfactory bulb (OB) brain region for **A**) CBD, **B**) 7-Carboxy cannabidiol (7CBD-COOH)

The PCA plot for CBD revealed visual similarities in the concentration of CBD in CTX, HP and STR regions, while the CBD distribution in the OB region was more widely dispersed, as illustrated by the scattered dot points. The overlapping ellipsoids suggest that the CBD distribution in the CTX, HP, STR regions is not significantly different. However, the wider spread of OB data points hints at a greater variability in CBD distribution within that region (Fig. 6A). In contrast, The PCA plot for 7COOH-CBD showed distinct distribution patterns. The CTX, HP, and STR regions exhibited similar 7COOH-CBD distribution, as evidenced by the clustering of their respective data points. Conversely, the OB region displayed a markedly different distribution, forming a separate cluster. This suggests that 7COOH-CBD is significantly more concentrated in the OB compared to the other regions. The non-overlapping ellipsoids for all brain regions further support the observation that 7COOH-CBD distribution varies significantly across these regions (Fig. 6B).

**Fig. 7:**
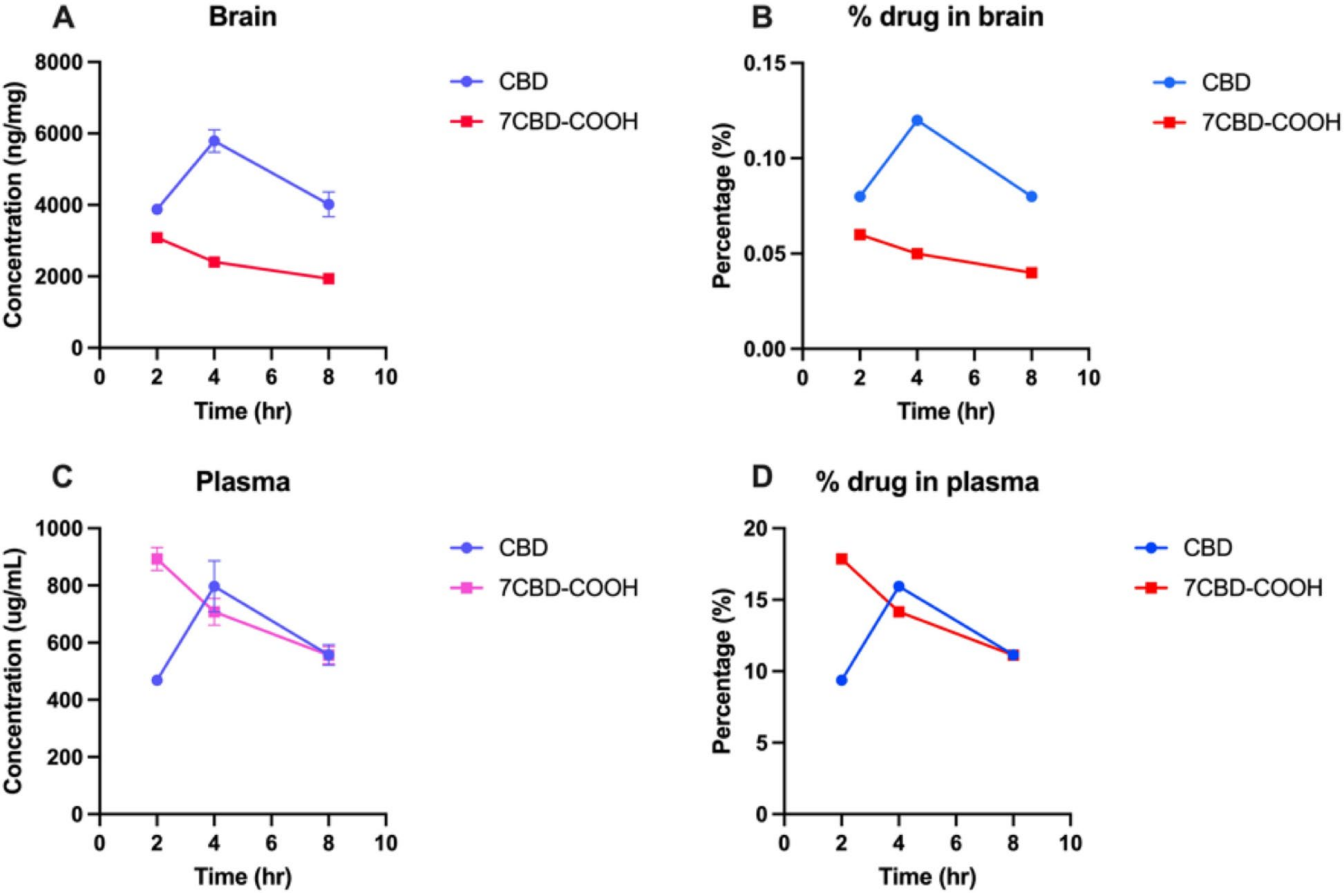
Connecting point graphs show changes in the concentration of CBD and its metabolite 7-Carboxy cannabidiol (7CBD-COOH) over 2, 4 and 8 h. **A** concentration in brain (**B**) percentage in brain (**C**) concentration in plasma (**D**) percentage in plasma. The percentage of the administered dose that reaches the brain is calculated as the sum of drug concentrations in hippocampus (HP), corpus striatum (STR), cerebral cortex (CTX), and olfactory bulb (OB) brain regions divided by the total dose administered (182 mg/kg)

The total concentration of CBD in the brain (HP + STR + CTX + OB) at 2, 4 and 8 h was 3879, 5788 and 4017 ng/mg, respectively and 7CBD-COOH was 3080, 2402 and 1937 ng/mg, respectively (Fig. 7A). This shows the maximum concentration of CBD attained in the brain at 4 h was 0.12% of the administered dose. The CBD is quickly metabolized to 7CBD-COOH by 2 h and continues to decrease over time (Fig. 7B). Similarly, in plasma, the concentration of CBD at 4 h reached 797 ug/mL (15.94% of the administered dose) while for 7CBD-COOH the concentration was highest at 2 h (893 ug/mL or 17.86% of the administered dose). The plasma levels indicate that as soon as CBD enters the system, it is rapidly metabolized, evidenced by peak levels of the metabolite by 2 h (Fig. 7C, D).

## Discussion and limitations

The purpose of this experiment was to design and prepare a hydrophilic CBD nanoformulation for IN administration to target the brain and to compare with the formulation containing Δ9THC alone. Our previous research measured CBD and Δ9THC (including THC's main metabolites) in various brain regions and organs. That study, which also used IN administration, found the lowest concentration of CBD in the liver (Upadhyay, et al., 2023). Therefore, we opted not to measure CBD levels in different organs for this experiment but in different brain regions and plasma. In the same study, the maximum concentration of Δ9THC was attained in the brain at 2 h while our current study revealed that the CBD concentrations in the brain peaked at 4 h (0.12%) whereas plasma concentrations peaked at 2 h (15.94%). The delayed peak of CBD in the OB, coupled with the rapid appearance of 7COOH-CBD in plasma and the OB, suggests efficient metabolic conversion and potential regional accumulation. While IN delivery can bypass the BBB to some extent, other barriers and transport mechanisms may influence drug distribution, for example the presence or activity of the cytochrome P450 (CYP) which is essential for converting CBD to 7COOH-CBD. The enzyme’s regional availability could also have contributed to the observed difference in 7COOH-CBD across brain regions. The IN route may be the better way to deliver the drugs to the brain, but this route may result in mild discomfort in mice indicated by transient rubbing of the nose with their paws. No adverse effects were observed. Others have reported that when CBD and Δ9THC were administered through an intraperitoneal (i.p.) route in 1:1 concentration, the concentrations of Δ9THC were higher in the brain compared to dosing with a formulation with a higher proportion of CBD (e.g., 1:5 or 1:10 Δ9THC: CBD combination)**.** CBD administration in combination with Δ9THC attenuates the psychotropic effects of Δ9THC, in part, by reducing its access to the brain (Reisdorph, et al., 2024). Our study reveals that IN administered THC and CBD peak at different time points in brain, they absorbed and metabolizes differently. However, to solidify these conclusion, future research should investigate both drugs concurrently in a single experiment. Another prior research suggests high exposure to Δ9THC (18% THC, 0.1% CBD) and CBD (13% CBD and 0.7% THC), results in lower accumulation in plasma and reduced cytokine and chemokine concentrations in the fetus’s brain and placenta in rats (Black, et al., 2023). But the same study doesn’t reveal the CBD concentrations in the brain of the fetus or female rats. A major limitation of our study is the use of just four male mice per group, potentially impacting the internal validity of the results. Given the sample size, the Principal Component Analysis (PCA) and heat map visualization should be interpretated as exploratory and indicative of potential trend, rather than definitive statistical conclusion. Other limitations of this study include the use of a single dose of CBD and a limited number of time points. Future investigations should explore potential sex differences in CBD absorption within the brain, dose-dependent pharmacokinetics of CBD and explore wider range of time points to fully characterize its distribution and metabolism. Additionally, investing in the effects of different formulations and delivery vehicles could help optimize IN CBD delivery.

## Conclusion

The results of this study demonstrate that 15.94% of the total intranasally administered CBD dose was detected in plasma, while 0.12% reached the brain. Although brain concentrations were lower than plasma levels, the successful detection and quantification of CBD and 7-COOH-CBD in distinct brain regions highlight the potential of intranasal delivery for direct nose-to-brain transport. Furthermore, the presence of 7-COOH-CBD in brain tissue suggests rapid metabolism of CBD within the central nervous system. Future work will focus on optimizing nanoformulations by incorporating mucoadhesive agents to enhance retention at the nasal epithelium and improve transport efficiency to the brain by adding long and short chain ligands in the nanoformulations to see region specific drug delivery to the brain, thereby advancing the therapeutic potential of CBD-based treatments for neurological disorders.

## Data Availability

The datasets used and/or analyzed during the current study are available from the corresponding author on reasonable request.

## Abbreviations

BBB: Blood–brain barrier
IN: Intranasal
CBD: Cannabidiol
7COOH-CBD: 7-Carboxy cannabidiol
Δ^**9**^THC: Delta-9-tetrahydro cannabinol
WT: Wild type
OB: Olfactory bulb
HP: Hippocampus
STR: Striatum
CTX: Cortex
LC–MS: Liquid chromatography mass spectrometry
NP: Nanoparticles
Ng: Nanogram
Mg: Milligram
Ug: Microgram
Kg: Kilogram
µL: Microliter
mL: Milliliter
PPM: Parts per Million
PTSD: Post-traumatic stress disorder
W/O: Water in oil
AVMA: American Veterinary Medical Association
SEM: Standard error of the mean
PCA: Principal component analysis
